# Supplementary material for: Design of precision therapeutics for a CKD risk allele by targeting Shroom3-Rock interaction
Source: Nat Commun. 2025 Dec 30;17:1086. doi: 10.1038/s41467-025-67854-7 (PMC12852734; doi:10.1038/s41467-025-67854-7)
Supplement: Supplementary file 1 — Supplementary Infomation [file 41467_2025_67854_MOESM1_ESM.pdf]

Design of precision therapeutics for a CKD risk allele by targeting Shroom3-Rock interaction

Authors:
Anand Reghuvaran PhD[1#]
Ashwani Kumar PhD[1#]
Qisheng Lin MD PhD[2]
Nallakandi Rajeevan PhD[3]
Khadija Banu PhD[1]
Zeguo Sun MS[4]
Hongmei Shi BS[1]
Gabriel Barsotti MD[1]
EM Tanvir PhD[1]
John Pell BS[1]
Sudhir Perincheri MD PhD[5]
Chengguo Wei PhD[4]
Bhavya Bharathan PhD[1]
Marina Planoutene PhD[4]
Anne Eichmann PhD[6]
Valeria Mas PhD[7]
Weijia Zhang, PhD[4]
Lloyd G Cantley MD[1]
Leyuan Xu PhD[1]
Bhaskar Das PhD*[8]
John Cijiang He MD PhD*[4]
Madhav C Menon MD*[1]

Affiliations:
1 Section of Nephrology, Department of Internal Medicine, Yale University School of Medicine, New Haven, CT, USA
2 Department of Nephrology, Renji Hospital, School of Medicine, Shanghai Jiao Tong University, Shanghai, 200127, China
3 Biomedical Informatics and Data Science, Yale University School of Medicine, New Haven, CT, USA
4 Division of Nephrology, Department of Medicine, Icahn School of Medicine at Mount Sinai, New York, NY, USA
5 Department of Pathology, Yale University School of Medicine, New Haven, CT, USA
6 Department of Molecular and Cellular Physiology, Yale University School of Medicine, New Haven, CT, USA
7 Surgical Sciences Division, Department of Surgery, School of Medicine, University of Maryland, Baltimore, MD, USA
8 University at Buffalo, SUNY Buffalo, Buffalo NY, USA.

Madhav C. Menon, MBBS MD FACP
Associate Professor
Nephrology, Medicine
Director of Research in Kidney Transplantation
Yale School of Medicine
New Haven, CT, USA-06519
203.737.4507
madhav.menon@yale.edu

John Cijiang He, MD PhD
Professor of Medicine and Pharmacological Sciences
Chief, Division of Nephrology, Medicine
Icahn School of Medicine at Mount Sinai
One Gustave L. Levy Place
New York, NY 10029
Cijiang.he@mssm.edu

Bhaskar Das, PhD
Professor of Pharmacology,
University of Buffalo State University of New York
Buffalo, NY 14203
bhaskard@buffalo.edu

Materials & Correspondence
All correspondence regarding data and material should be addressed to madhav.menon@yale.edu

Supplementary Figures 1-8

Figure-S1

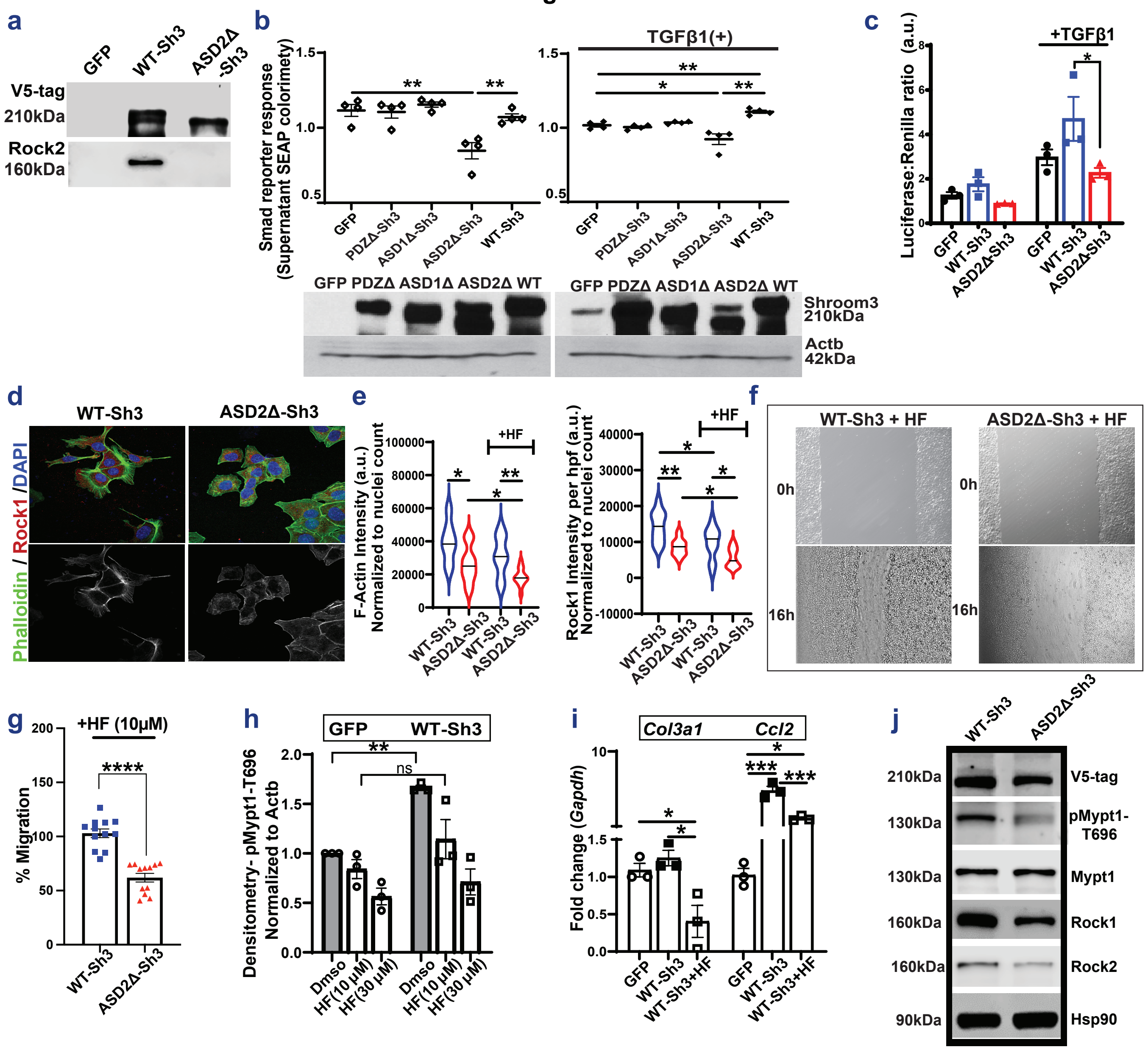

Figure-S1 <u>ASD2Δ-Sh3-overexpression reduces ROCK activation in tubular cells and fibroblasts;</u> (**a**) Representative immunoblots of SHROOM3 and ROCK2 from lysates immunoprecipitated from HEK-293T cells overexpressing WT-Sh3 or ASD1Δ-Sh3 using anti-V5 beads (n=2 experiments). **(b)** Dot plots (top) depict the relative Smad reporter response of TGFβ- or vehicle-treated HEK-293T cells overexpressing different Shroom3-variant constructs in the presence or absence of TGFβ. Reporter response is measured as the level of Secreted embryonic alkaline phosphatase (SEAP) produced in the culture supernatant (n=4 experiments; Unpaired T test p-values in Source data). Representative immunoblots (bottom) of SHROOM3 and ACTB using lysates from these cells . (**c**) Bar graph representing Luciferase:Renilla ratio of Smad-promoter-responder constructs to assess TGFβ signaling, comparing WT-Sh3 or ASD2Δ-Sh3 overexpressing HEK-293T cells with/without TGFβ (5ng/mL) (n=3 experiments; One way ANOVA & Bonferroni-Holm's post test used). (**d**) Representative fluoromicrographs of immunostaining of Rock1 and F-Actin in WT-Sh3 or ASD2Δ-Sh3 overexpressing IMCD cells in the presence/absence of Fasudil (HF). (**e**) Violin plots quantify the mean fluorescence intensities of F-Actin and Rock1 (normalized to the total nuclear count per hpf from 2 experiments; Mann-Whitney test used). Partial cells abutting field margins were excluded **(f)** Representative phase contrast micrographs (4x) of scratches at 0h and 16 h of IMCD cells over-expressing WT-Sh3/ ASD2Δ-Sh3 treated with HF (10µM) with (**g**) the corresponding bar graph quantifying the migrated area per field of ASD2Δ-Sh3 IMCD at 16hrs (normalized to WT-Sh3 fields) obtained from 3 fields/well, & 3 wells/biological replicate (Mann-Whitney test P<0.0001). **(h)** Bar graphs depicting the densitometry measurements for pMypt1:Actb in WT-Sh3-IMCD with/without HF treatments (n=3 experiments; Unpaired Welch's T tests). (**i**) Relative gene expression of *Col3a1*

& *Ccl2*, before or after HF treatment in WT-Sh3-IMCD. GFP control group served as reference (n=3 experiments; One way ANOVA & Holm-Šídák's tests) (**j**) Representative immunoblots of V5-tag (Shroom3)/pMypt1/Mypt1/ Rock1/Hsp90 in 3T3 fibroblasts. [Line and whiskers= mean ± SEM; Two-tailed p-values denoted as *p < 0.05, **p < 0.01, ***p < 0.001; Hpf=high power field. Exact p-values of significant tests shown are in source data. Blue symbols depict WT-Sh3 and red depict ASD2Δ-Sh3 expressing cells, while black depict GFP controls].

Figure-S2

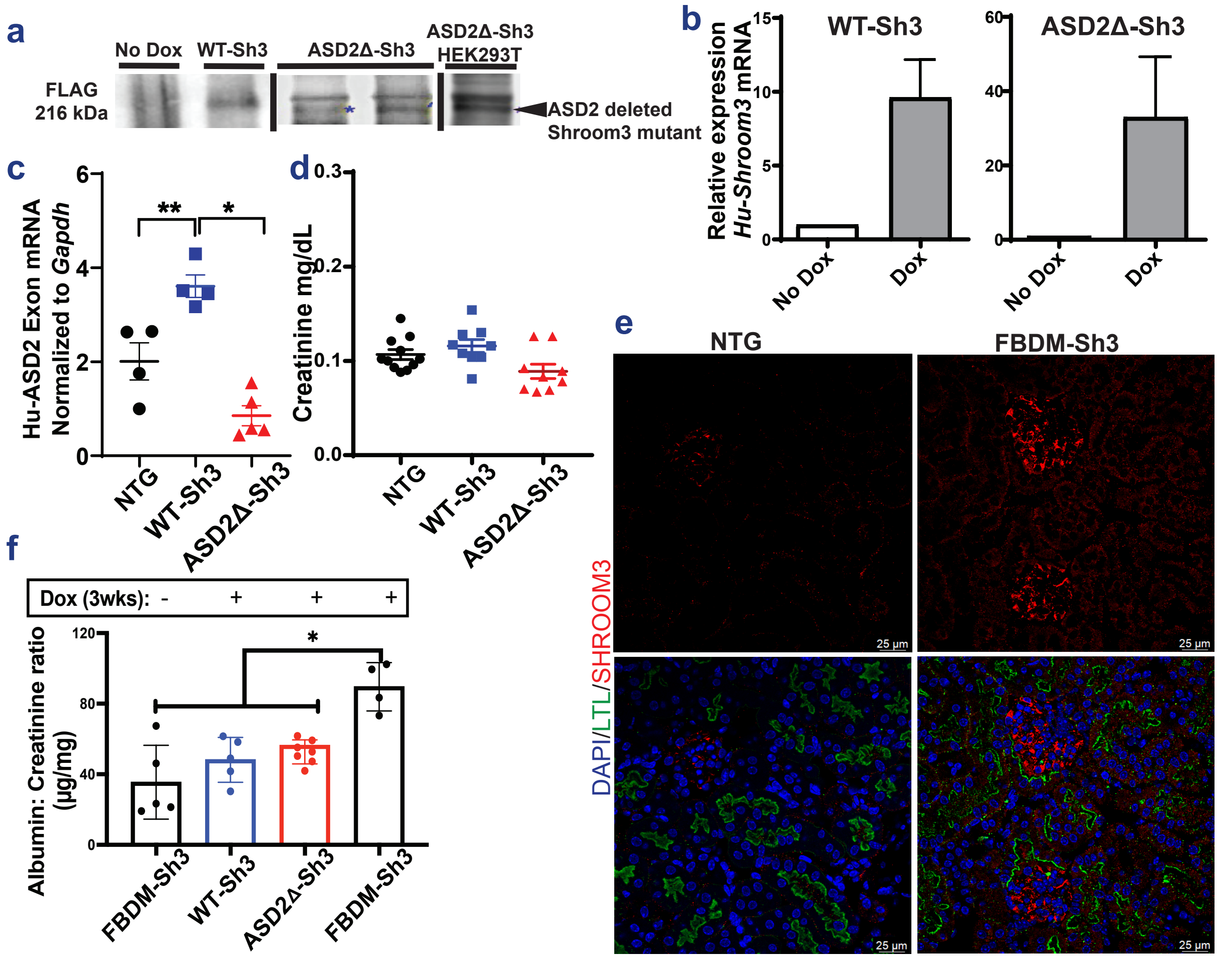

Figure-S2 **Generation and phenotyping of global, ASD2Δ-Sh3 overexpression mice;** (**a**) Immunoblot of FLAG tag to confirm overexpression of SHROOM3 *in vivo.* (Arrowhead shows the lower molecular weight ASD2-deleted Shroom3 mutant band; the blots are from different gels but run parallelly) (**b**) Bar graphs denoting relative mRNA expressions of *SHROOM3* in TG mice post DOX-induction; WT-Sh3 (n=2) & ASD2Δ-Sh3 (n=4). (**c**) Dot plot of mRNA levels of *SHROOM3* exon harboring the ASD2-domain in WT-Sh3 mice vs. ASD2Δ-Sh3, post DOX-induction (NTG vs. WT-Sh3 vs. ASD2Δ-Sh3; n=4 vs. 4 vs. 5 mice). Data was analyzed by One-way ANOVA with Tukey's post hoc tests (NTG vs. WT-Sh3; p= 0.008, WT-Sh3 vs. ASD2Δ-Sh3; p=0.037, NTG vs. ASD2Δ-Sh3; p=0.0001) (**d**) Dot plot of baseline serum creatinine levels in the DOX-treated mice analyzed using One-way ANOVA with Tukey's post hoc tests (NTG vs. WT-Sh3 vs. ASD2Δ-Sh3; n=11 vs. 9 vs. 9 mice). (**e**) Representative fluoromicrographs of SHROOM3/LTL (Red/Green) immunostaining confirming overexpression in FBDM-Sh3 mice post DOX-induction (n=3 each). (**f**) Bar graph representing urine albumin to creatinine ratios in FBDM-Sh3 mice compared to other Shroom3-TG mice and Mice without DOX induction (FBDM-Sh3-No DOX vs. WT-Sh3 vs. ASD2Δ-Sh3 vs. FBDM-Sh3-DOX; n=5 vs. 5 vs. 7 vs. 4 mice; Mann-Whitney T tests). [Line and whiskers indicate mean ± SEM; Two-tailed p-values denoted as *p < 0.05, **p < 0.01; For all graphs exact p-values of significant tests are also given in the source data. In 2c, 2d and 2f, blue symbols depict WT-Sh3 and red depict ASD2Δ-Sh3 mice, while black symbols depict NTG in the former two graphs and FBDM-Sh3 mice in the latter; DOX- Doxycycline, FBDM-Sh3- Fyn binding domain mutant Shroom3].

Figure-S3

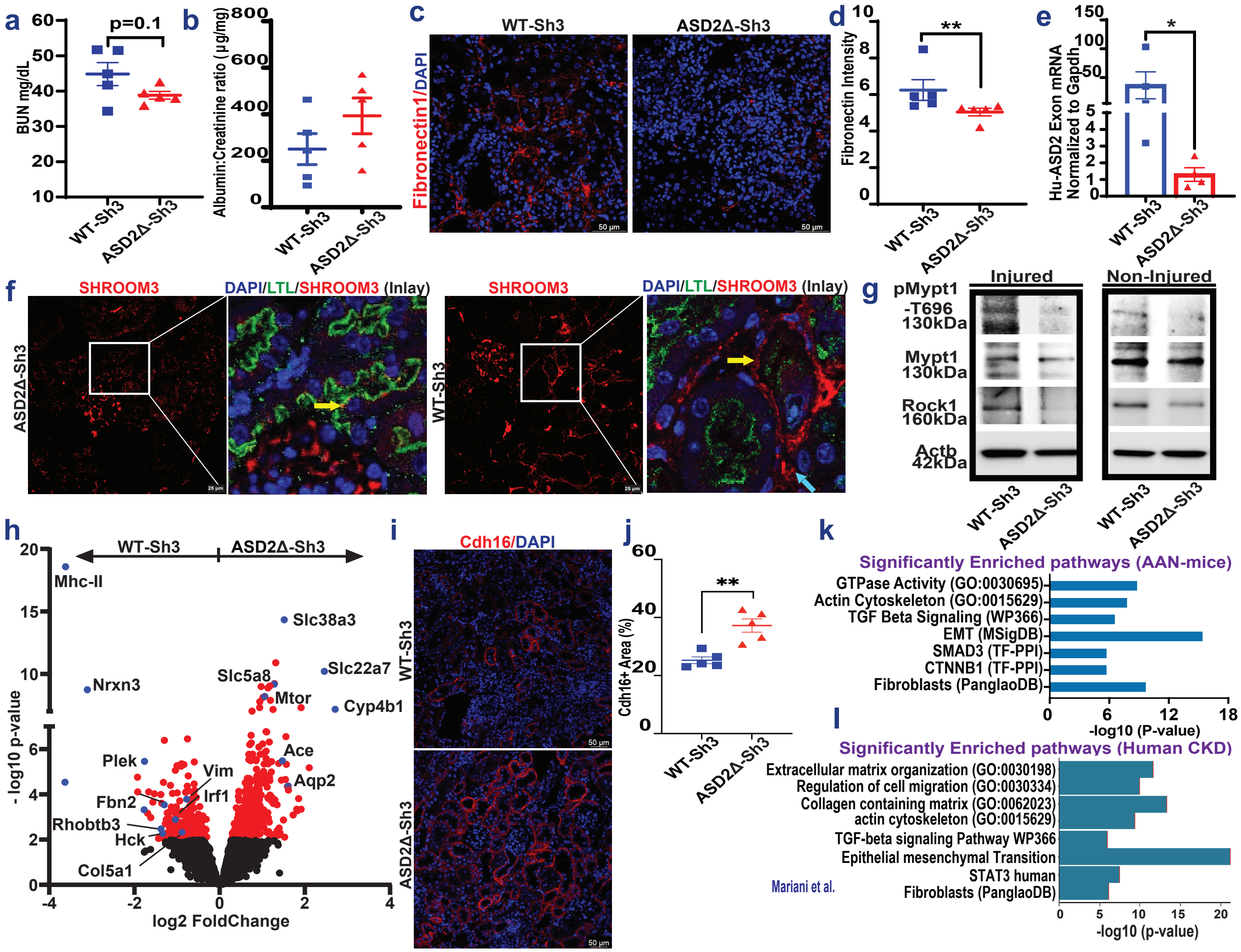

Figure-S3 Global ASD2Δ-Sh3 mice showing reduced TIF vs WT-Sh3 overexpression in AAN model; Dot plots depict (**a**) the BUN levels (Unpaired T test p=0.1) and (**b**) Urine albumin to creatinine ratios in the AAN mice in WT-Sh3 and ASD2Δ-Sh3 (n=5 each; Mann-Whitney p=0.15) groups. (**c**) Fibronectin immunofluorescence (IF) showing red color positivity (20X), merged with DAPI (blue nuclei), bar graph (**d**) for IF quantification indicating more fibrosis (Mann-Whitney p=0.008) in WT-Sh3 as compared to ASD2Δ-Sh3 mice (n=5 each). (**e**) Bar graph denoting relative mRNA expressions of *Shroom3* exon harboring the ASD2-domain in AAI-injured kidneys of WT-Sh3 vs ASD2Δ-Sh3 (n=4 each; Mann-Whitney p=0.029). (**f**) Representative fluoromicrographs (40X and inlay) of SHROOM3/LTL staining in AAN-kidney sections for global overexpression mice showing Shroom3 tissue localization post-injury (ASD2Δ-Sh3 vs WT-Sh3; n=3 each). (**g**) Immunoblots of pMypt1, Mypt1, Rock1, and Actb with AAN-injured vs non-injured kidney lysates (WT-Sh3 vs ASD2Δ-Sh3; n=2 each). (**h**) Volcano plot representing significant DEGs identified by DESeq from GRCh38 alignment for RNA sequencing dataset from AAN mice (WT-Sh3 vs ASD2Δ-Sh3; n=4 each). The count data were analyzed by DESeq2 using a negative binomial generalized linear model. The top 500 upregulated DEGs in WT-Sh3 AAN-kidneys are shown by fold change (p<0.05). (**i**) IF staining for KSP-Cadherin (20X) showing red color positivity co-stained with DAPI (blue nuclei), and (**j**) bar graph showing percentage area of KSP-Cadherin positive tubules indicating better tubular preservation in ASD2Δ-Sh3 as compared to WT-Sh3 (n=5 each; Mann-Whitney p=0.008). (**k**) Bar graphs representing the significantly enriched pathways identified by EnrichR analysis of AAN-Mice. (**l**) co-expression analyses of enriched genes from another tubulo-interstitial transcriptome of human CKD cohorts within Nephroseq, which correlated with Shroom3 expression (adjusted p-values of Fisher's exact tests- two-tailed; See reference 34). [Line and whiskers indicate mean ± SEM; Two-tailed p-values denoted as *p < 0.05, **p < 0.01; For all graphs exact p-values of significant tests are also given in the source data. In S3a, b, d, e, j, blue symbols depict WT-Sh3 while red depict ASD2Δ-Sh3 mice; In S3h red symbols depict significant genes vs. non-significant black ones].

Figure-S4

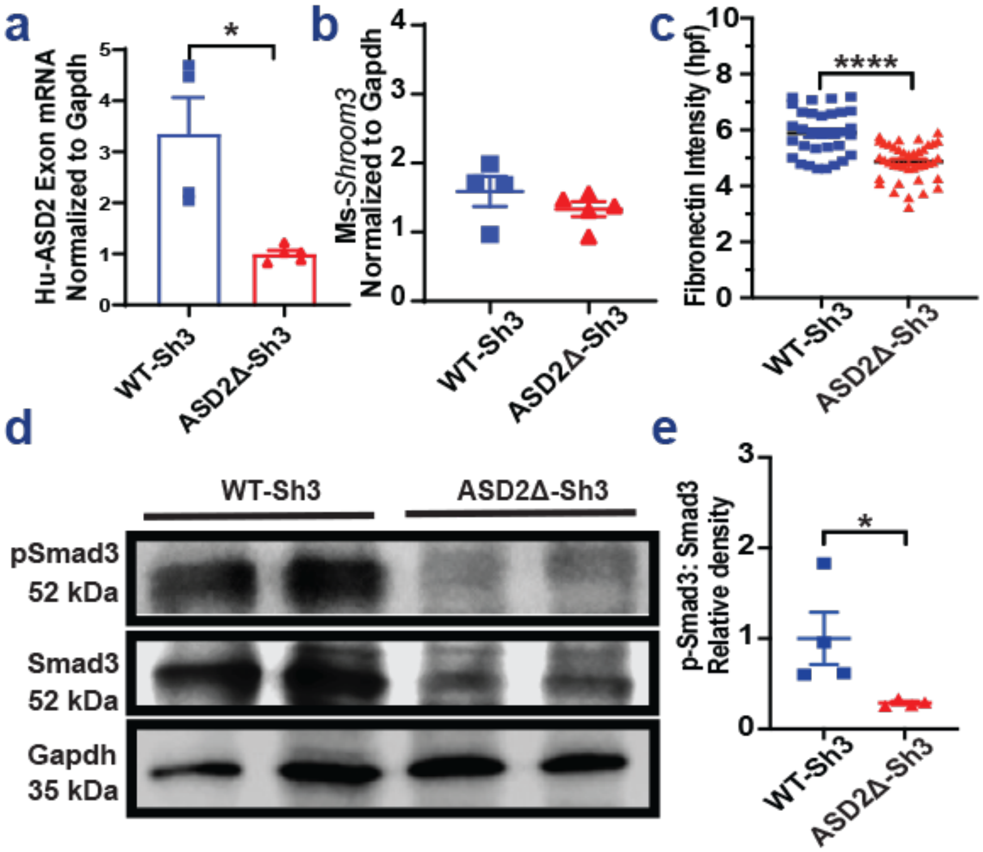

Figure-S4 Global ASD2Δ-Sh3 showing reduced TIF vs WT-Sh3 overexpression in UUO model; (**a**) Bar graph representing the relative mRNA expression using ASD2-domain specific primers in UUO kidneys of CAGSrtTA-WT-Sh3 vs. ASD2Δ-Sh3 mice (n=4 vs. 5; Mann-Whitney p=0.016). **(b)** Dot plot denoting relative mRNA expressions of mouse endogenous Shroom3 in UUO kidneys of WT-Sh3 vs. ASD2Δ-Sh3 mice (n=4 vs. 5; Mann-Whitney p=0.19). (**c**) Dot plots show quantification of immunofluorescence staining for Fibronectin by high power field (8hpf/kidney) in UUO kidneys of WT-Sh3 vs. ASD2Δ-Sh3 mice (n=4 vs. 5; Mann-Whitney p<0.0001). **(d)** Representative immunoblots of whole kidney lysates from UUO kidneys of WT-Sh3 or -ASD2Δ-Sh3 mice probed for pSmad3/Smad3/Gapdh, and (**e**) dot plots show respective quantification of pSmad3:Smad3 ratio by densitometry (n=4 vs. 5; Mann-Whitney p=0.029). [Line and whiskers indicate mean ± SEM; Two-tailed p-values denoted as *p < 0.05, ****p < 0.0001; For all graphs exact p-values are also given in the source data and blue symbols depict WT-Sh3 and red depict ASD2Δ-Sh3 mice].

Figure-S5

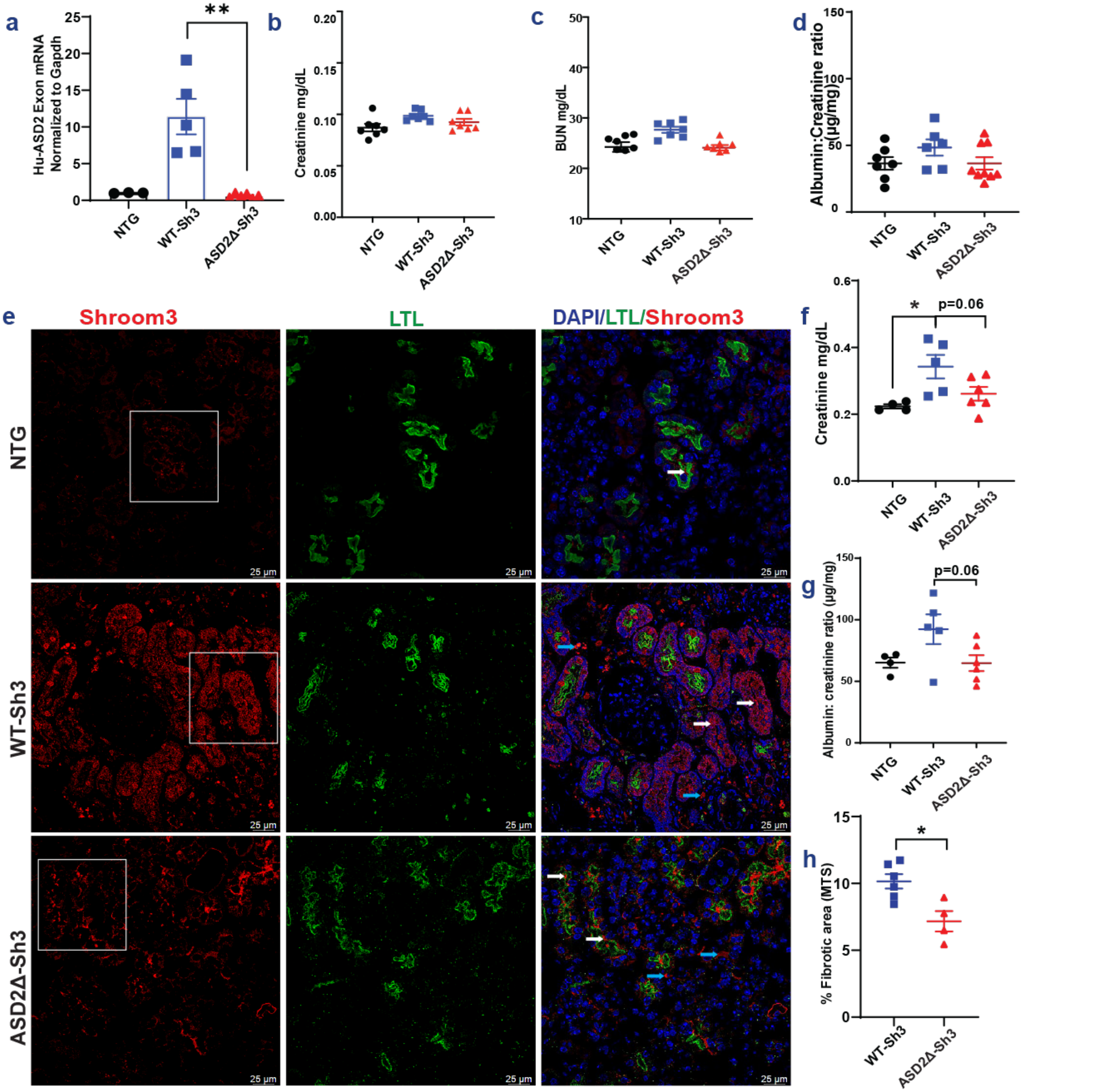

Figure-S5 T<u>ubular-specific ASD2Δ-Sh3 mice showing reduced TIF vs WT-Sh3;</u> (**a**) Bar graph representing the relative mRNA expressions by qPCR using ASD2-domain specific primers in DOX-treated Pax8-rtTA/WT-Sh3, - ASD2Δ-Sh3 or NTG mice (n=5 vs. 6 vs. 3 respectively; Kruskal-Wallis with Dunn's tests- WT-Sh3 vs ASD2Δ-Sh3, p=0.003). **(b-d)** Dot plot showing **(b)** serum creatinine, (**c**) BUN levels, & **(d)** urine albumin: creatinine ratios in adult DOX-treated Pax8-rtTA mice and non-transgenic mice (4 weeks DOX = Doxycycline chow + water 200mg/L). For **(b)** and **(c)** n=7 mice/group and for **(d)** NTG (n=7) vs. WT-Sh3 (n=6) vs. ASD2Δ-Sh3 (n=9), and One-way ANOVA with Tukey's tests were used (p-values in source data). (**e**) Representative fluoromicrographs of SHROOM3/LTL immunostaining in AAN kidneys of Pax8-rtTA- WT-Sh3/ASD2Δ-Sh3 or NTG animals (n=3 each). Dot plots of (**f**) serum creatinine levels (**g**) urine albumin to creatinine ratios in the AAN mice at 9-wks (NTG vs. WT-Sh3 vs. ASD2Δ-Sh3; n=4 vs. 5 vs. 6 respectively; p=0.006 for both WT-Sh3 vs. ASD2Δ-Sh3). Both data were analyzed by One-way ANOVA with Tukey's tests (p-values in source data). **(h)** As shown in Fig 5, a second model of TIF i.e. UUO was created in Pax8-rtTA-WT-Sh3/ASD2Δ-Sh3 (n=6 vs 4). Dot plots compare blue-stained area (10 hpf per animal) in Trichrome-stained sections each UUO kidney (WT-Sh3 vs. ASD2Δ-Sh3; Mann-Whitney p = 0.019). [Line and whiskers indicate mean ± SEM; Mann-Whitney T-test, Two-tailed p-values denoted as *p < 0.05; For

all graphs exact p-values of significant tests are also given in the source data; blue symbols depict WT-Sh3, red depict ASD2Δ-Sh3 and black depicts NTG mice].

Figure-S6

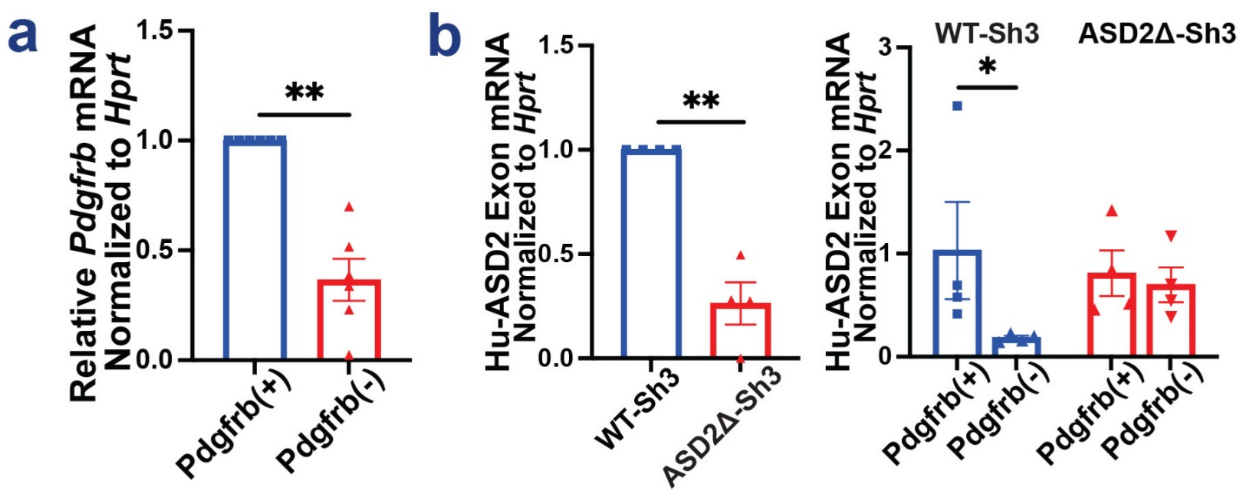

Figure-S6 **Fibroblast-specific ASD2Δ-Sh3 mice showing similar TIF to WT-Sh3 overexpression in UUO model;** Pdgfrb+/Pdgfrb- cells were isolated from Pdgfrb-rtTA-WT-Sh3/ASD2Δ-Sh3 mice, and qPCR was performed on extracted RNA. Bar graph depicting (**A**) *Pdgfrb* mRNA expressions in Pdgfrb+ (blue) vs. Pdgfrb- (red) cells (n= 3 mice from each line, 2 replicates- one isolation per kidney of each mouse; paired T test p= 0.001). (**B**) Bar graphs depicting *Shroom3*-ASD2 domain (domain specific primers) mRNA expressions in Pdgfrb+ cells to compare the two lines- WT-Sh3 (blue) vs. ASD2Δ-Sh3 (red) on left panel, while the right panel shows *SHROOM3*-ASD2 domain expression in Pdgfrb+(blue) vs. Pdgfrb- (red) cells within each respective line (n= 4 mice from each line). For *SHROOM3*-ASD2 domain expression between WT-Sh3 vs. ASD2Δ-Sh3, paired T test p= 0.005 and for Pdgfrb+ vs. Pdgfrb- in WT-Sh3 line, Friedman's ANOVA with Dunn's test p= 0.012. [Line and whiskers indicate mean ± SEM; unpaired T-test, Two-tailed p-values denoted as *p < 0.05, **p < 0.01].

Figure-S7

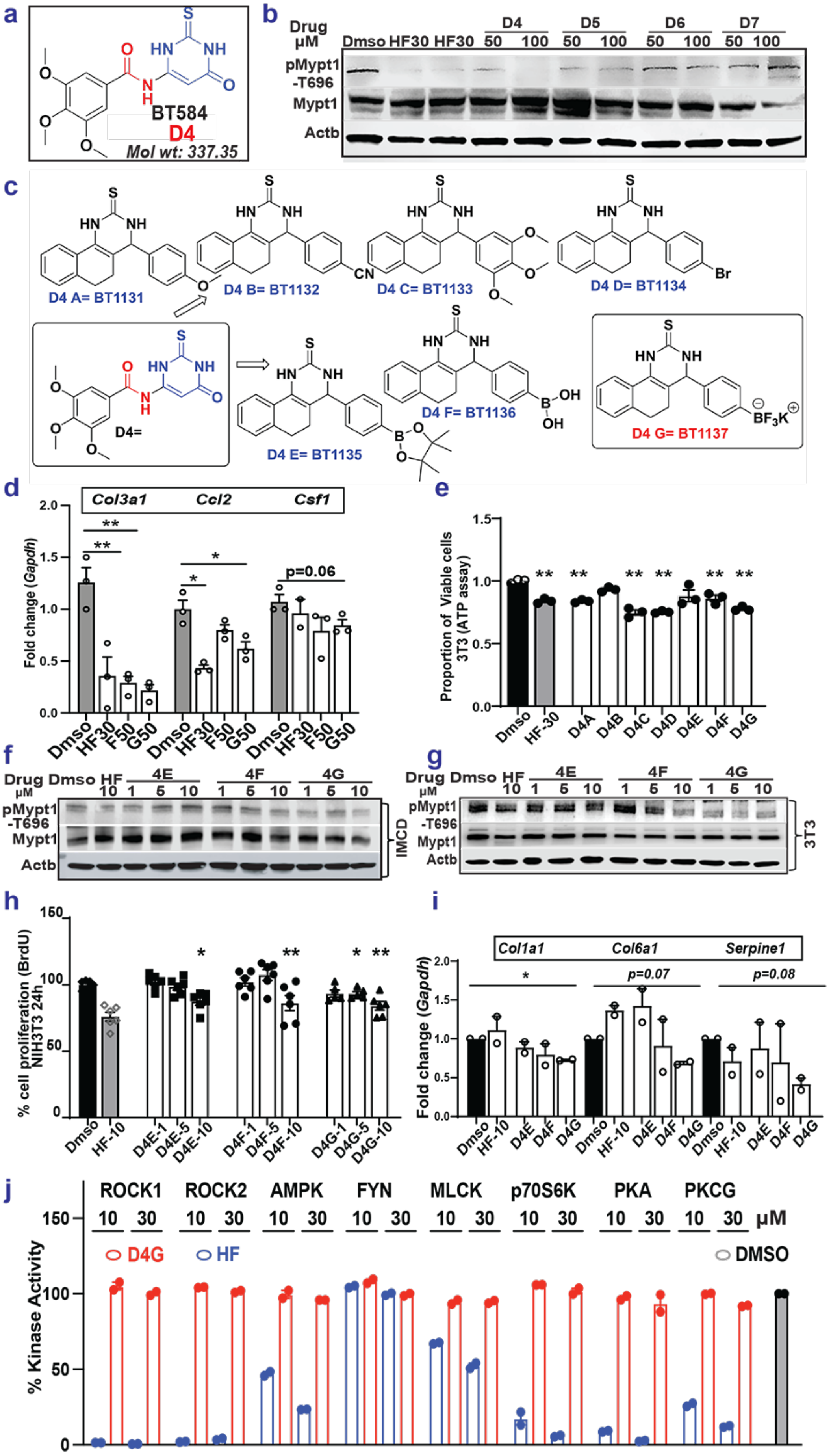
a
BT584
D4
Mol wt: 337.35
b
Drug μM
Dmso HF30 HF30 D4 50 100 D5 50 100 D6 50 100 D7 50 100
pMypt1 -T696
Mypt1
Actb
c
D4 A= BT1131
D4 B= BT1132
D4 C= BT1133
D4 D= BT1134
D4=
D4 E= BT1135
D4 F= BT1136
D4 G= BT1137
d
Col3a1 Ccl2 Csf1
Fold change (Gapdh)
p=0.06
Dmso HF30 F50 G50
e
Proportion of Viable cells 3T3 (ATP assay)
Dmso HF-30 D4A D4B D4C D4D D4E D4F D4G
f
Drug μM Dmso HF 10 4E 1 5 10 4F 1 5 10 4G 1 5 10
pMypt1 -T696
Mypt1
Actb
IMCD
g
Drug μM Dmso HF 10 4E 1 5 10 4F 1 5 10 4G 1 5 10
pMypt1 -T696
Mypt1
Actb
3T3
h
% cell proliferation (BrdU) NIH3T3 24h
Dmso HF-10 D4E-1 D4E-5 D4E-10 D4F-1 D4F-5 D4F-10 D4G-1 D4G-5 D4G-10
i
Col1a1 Col6a1 Serpine1
Fold change (Gapdh)
p=0.07
p=0.08
Dmso HF-10 D4E D4F D4G
j
ROCK1 ROCK2 AMPK FYN MLCK p70S6K PKA PKCG
10 30 μM
D4G
HF
DMSO
% Kinase Activity

Figure-S7 **Design and Synthesis of Shroom3-Rock interaction inhibitors to inhibit ROCK activity;** (**a**) Structure of compound BT584 (D4) is shown. (**b**) Representative immunoblots for pMypt1/Mypt1/Actb from HEK293T cells treated with D4-D7 at 50-100μM in HEK293T cell lysates. (**c**) Chemical structures of the compounds D4A-D4G derived from the parent compound BT584. (**d**) Bar graph representing the relative mRNA expressions of pro-fibrotic and cytokine markers in WT-Sh3-IMCD cells treated with 50μM D4F and D4G (n=3 experiments; Unpaired T tests). (**e**) Bar graph depicting the toxicity assay on WT-Sh3-3T3 cells 24h post treatments with 50μM D4A-D4G (One-way ANOVA with Dunnett's tests). (**f-g**) Representative immunoblots from WT-Sh3 overexpressing cells treated with 1, 5 and 10μM D4E-D4G for pMypt1/Mypt1/Actb from **(f)** IMCD- & **(g)** from 3T3- cell lines. Bar graphs representing (**h**) WT-Sh3-3T3 proliferation on D4E-D4G (1-10μM) treatments (n=6 experiments; One-way ANOVA with Dunnett's tests) and **(i)** the relative mRNA expressions of pro-fibrotic markers with D4E-D4G (10μM) treated WT-Sh3-3T3 cells (n=2 experiments; two-tailed paired T tests). **(j)** Bar graph depicting the percent activities of kinases on incubation with 10μM D4G vs. 30μM Fasudil (HF); n=2 replicates. [Line and whiskers indicate mean ± SEM; Unpaired T-test, except in non-normal data distributions, Two-tailed p-values denoted as *p < 0.05, **p < 0.01; For all graphs exact p-values are also given in the source data].

Figure-S8

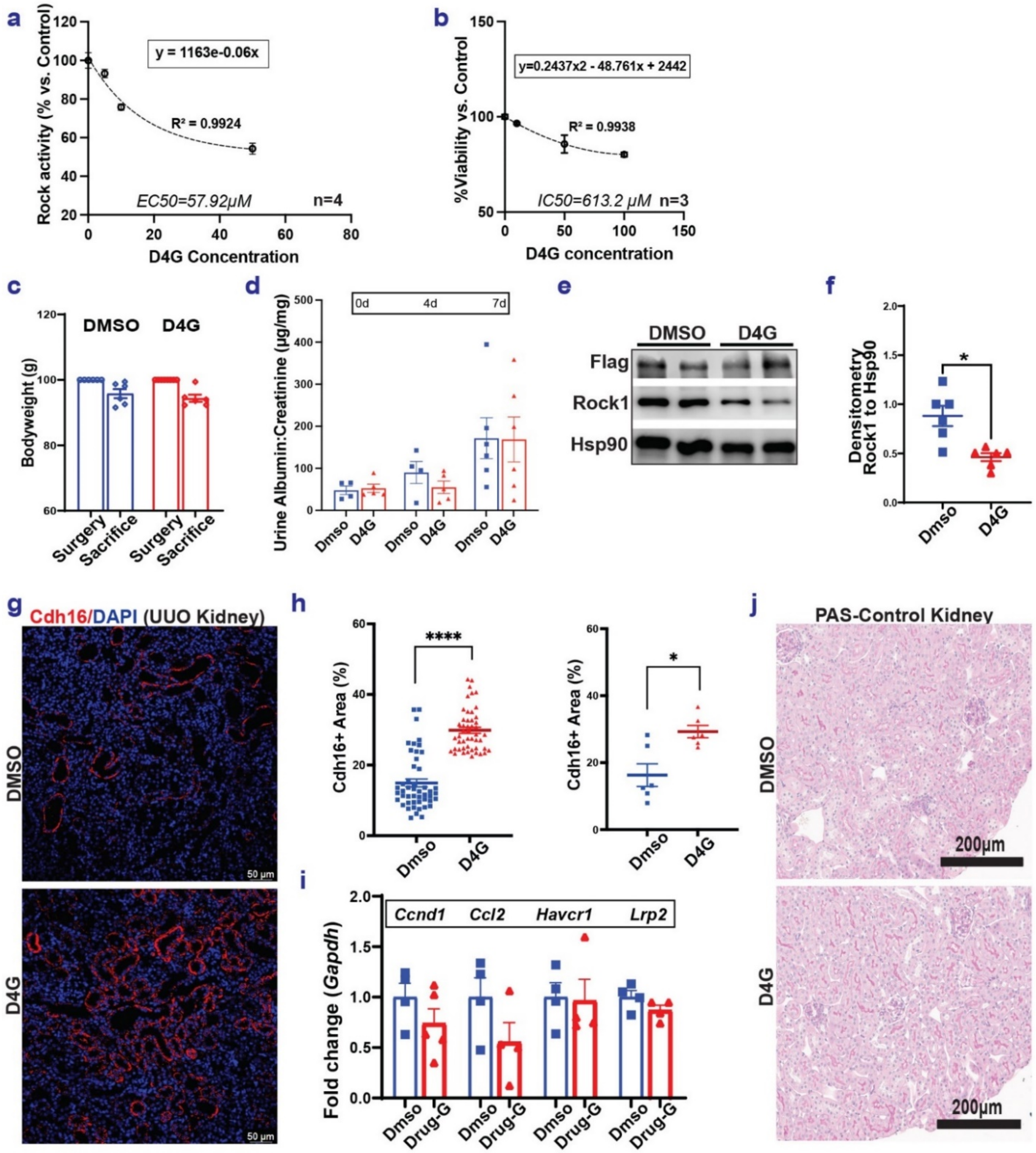

Figure-S8 **<u>D4G ameliorated TIF progression in Tubular-Specific WT-Sh3 overexpression mice;</u>** TIF was induced by UUO in Pax8-rtTA/WT-Sh3 mice followed by treatment with drug D4G or vehicle (5% Dmso in Corn oil) (n=6 each). Plots of non-linear lines-of-fit depicting **(a)** the EC50 for Rock inhibition (n=4 experiments) and **(b)** the IC50 for cell viability in WT-SH3-IMCD cells by the drug D4G (n=3 experiments). **(c-d)** Bar graphs compare **(c)** the % body weights of mice (DMSO/D4G n=6 each) on the day of UUO surgery and the day of sacrifice (DMSO vs. D4G at day-7; n=6 each; Unpaired T test p= 0.417) and **(d)** the urine albumin to creatinine ratios of D4G or DMSO treated mice (n=6 each at day-7; Mann-Whitney tests). **(e)** Representative immunoblots from UUO-kidney lysates for Flag, Rock1 and Hsp90 and **(f)** the dot plot depicting the quantification of relative Rock1 levels (n=6 mice/group; Mann-Whitney p=0.004). **(g)** Representative fluoromicrographs of Cdh16 (D4G vs. DMSO) and **(h)** the quantification of percent Cdh16 positive area per hpf (8hpf/kidney; Mann-Whitney p<0.0001), and per UUO kidney (D4G vs. DMSO; n=6 each; Mann-Whitney p=0.041). **(i)** Bar graphs show selected mRNA transcripts from non-UUO (uninjured) kidney lysates to show absence of any significant injury induced by D4G vs DMSO (Mann-Whitney tests used; n=4 each for *Ccl2* {p=0.343}, *Havcr1* {p=0.886} and *Lrp2* {p=0.2}; n=4 vs. 5 for *Ccnd1*, p=0.486). **(j)** Representative photomicrographs (20X) of PAS-stained non-UUO kidney sections (D4G vs DMSO). [Line and whiskers indicate mean ± SEM; Two-tailed p-values denoted as *p < 0.05, **p < 0.01; For all graphs exact p-values are also given in the source data; blue symbols depict DMSO, red depict D4G]. [EC50= Effective concentration-50, IC50= Inhibitory concentration-50, hpf= high power field, PAS = Periodic acid-Schiff].
