# Supplementary material for: Design of precision therapeutics for a CKD risk allele by targeting Shroom3-Rock interaction
Source: Nat Commun. 2025 Dec 30;17:1086. doi: 10.1038/s41467-025-67854-7 (PMC12852734; doi:10.1038/s41467-025-67854-7)
Supplement: Supplementary file 2 — Description of Additional Supplementary Files [file 41467_2025_67854_MOESM2_ESM.pdf]

Description of Additional Supplementary Files

Title: Supplementary Dataset S1
Description:
1a: Table showing differential gene expression after aligning reads with human orthologs (GRCh38) transcriptome.
1b: Table showing differential gene expression after aligning reads with mouse (mm39) orthologs transcriptome
1c: List of primers used for PCR amplification

Title: Supplementary Dataset S2
Description:
Synthesis of the small molecule BT-1137
NMR analysis of the small molecule BT-1137
