# Supplementary material for: Design of precision therapeutics for a CKD risk allele by targeting Shroom3-Rock interaction
Source: Nat Commun. 2025 Dec 30;17:1086. doi: 10.1038/s41467-025-67854-7 (PMC12852734; doi:10.1038/s41467-025-67854-7)
Supplement: Supplementary file 4 — Supplementary Dataset S2 [file 41467_2025_67854_MOESM4_ESM.pdf]

Supplementary Dataset 2

Synthesis of the small molecule BT-1137

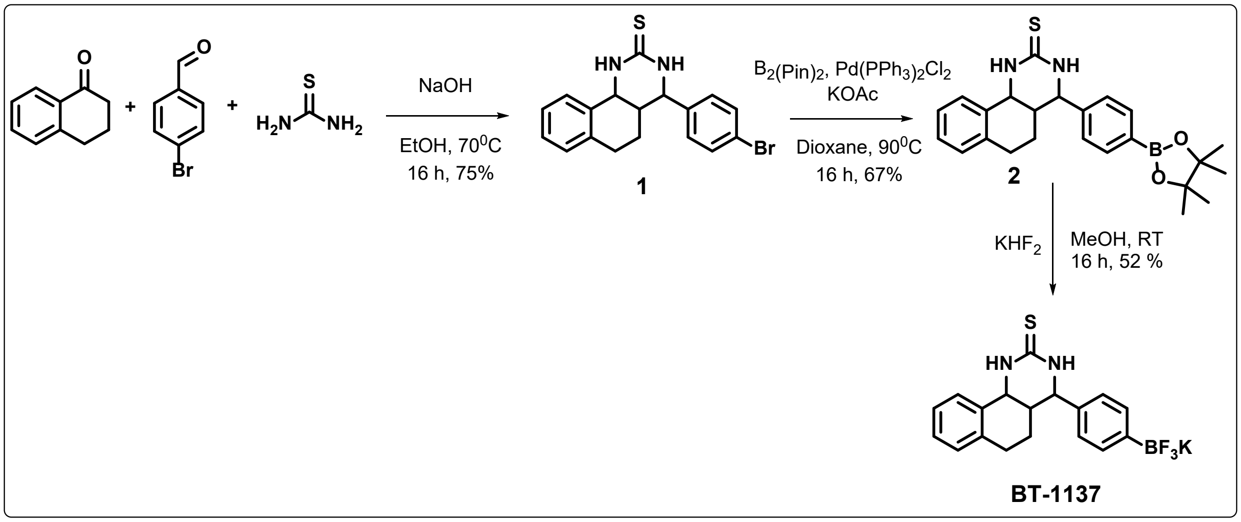

Step 1:

To a stirred solution of ketone (1 equ) in EtOH (5 mL), 4-bromo benzaldehyde (1 equ), thio-urea (1 equ), and NaOH (1 equ) were added and heated at 70°C. After 15 min, yellow solid was thrown out from the reaction mixture. The heating was continued for 16 h, the yellow solid was slowly dissolved, and a white solid was precipitated from the reaction mixture. The white solid was filtered and washed twice with cold ethanol to afford compound **1** in 75% yields.

Step 2:

To a stirred solution of compound **1** (1 equ) in dioxane (3 mL), B_2Pin_2 (1.5 equ), KOAc (2 equ), and $Pd_2(PPh_3)_2Cl_2$ (0.2 equ) were added and heated at 90°C. The reaction mixture was stirred at 90°C for 16 h under a nitrogen atmosphere. Reaction progress was monitored by TLC. After completion of the reaction, the reaction mass was allowed to cool at ambient temperature and the reaction mixture was filtered through celite. The reaction was extracted with EtOAc and the crude was purified by column chromatography to afford compound **2** in 67% yields.

Step 3:

To a stirred solution of compound **2** (1 equ) in MeOH (3 mL), and KHF_2 (2 equ) were added and stirred at RT for 16 h. Reaction progress was monitored by TLC. After completion of the reaction, the reaction mass was filtered and washed with acetone and ether to afford compound **BT-1137** in 52% yields.

NMR analysis of the small molecule BT-1137

PC-C-01

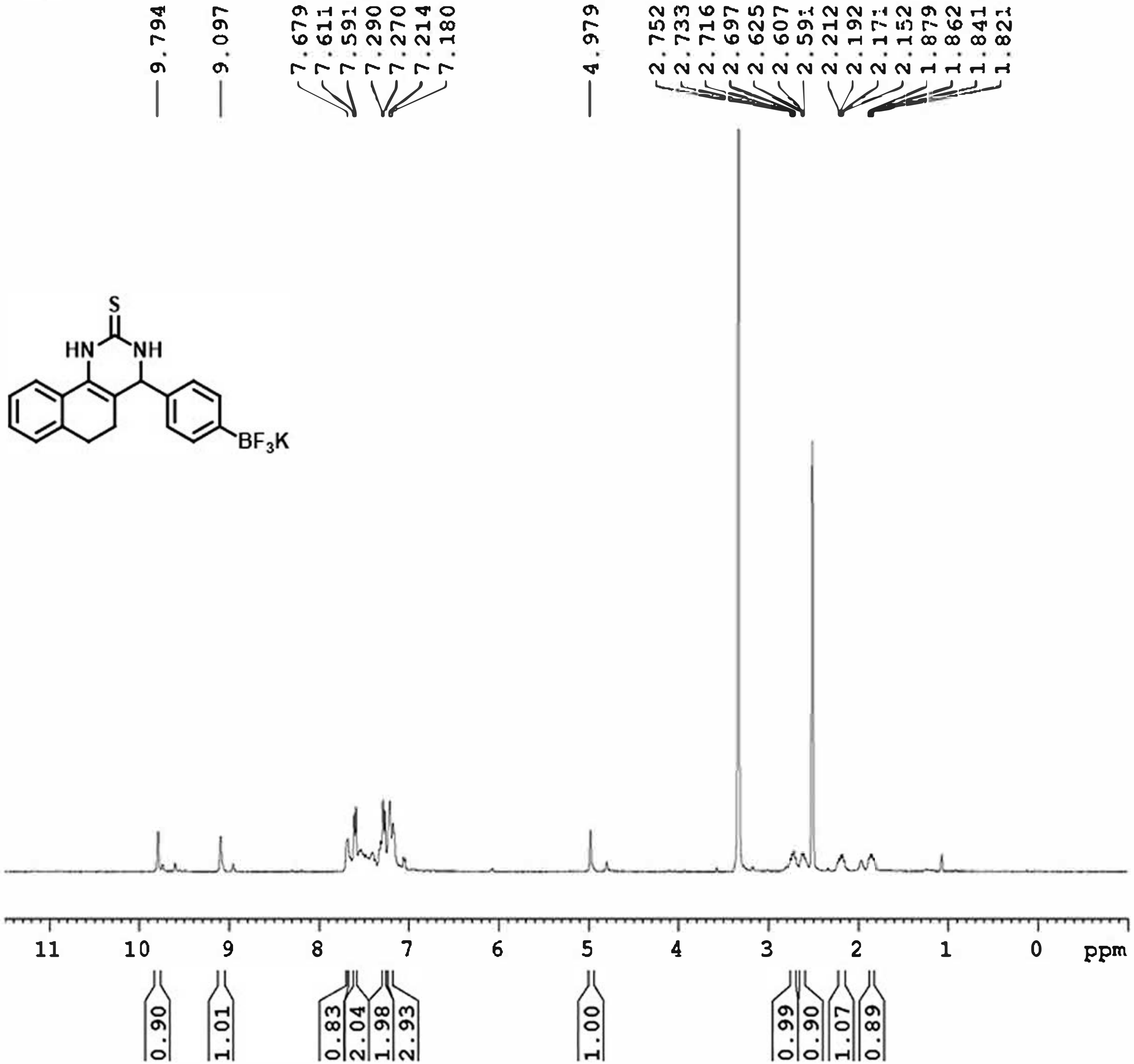

Current Data Parameters

NAME	Aug16-2023
EXPNO	1
PROCNO	1

F2 - Acquisition Parameters

Date_	20230816
Time	11.11 h
INSTRUM	Avance Neo 400 Nanobay
PROBHD	2163739_0311 (
PULPROG	zg30
TD	65536
SOLVENT	DMSO
NS	16
DS	2
SWH	8196.722 Hz
FIDRES	0.250144 Hz
AQ	3.9976959 sec
RG	101
DW	61.000 usec
DE	13.89 usec
TE	298.0 K
D1	1.00000000 sec
TD0	1
SFO1	400.1474709 MHz
NUC1	1H
P0	2.67 usec
P1	8.00 usec
PLW1	20.98500061 W

F2 - Processing parameters

SI	65536
SF	400.1450000 MHz
WDW	EM
SSB	0
LB	0.30 Hz
GB	0
PC	1.00

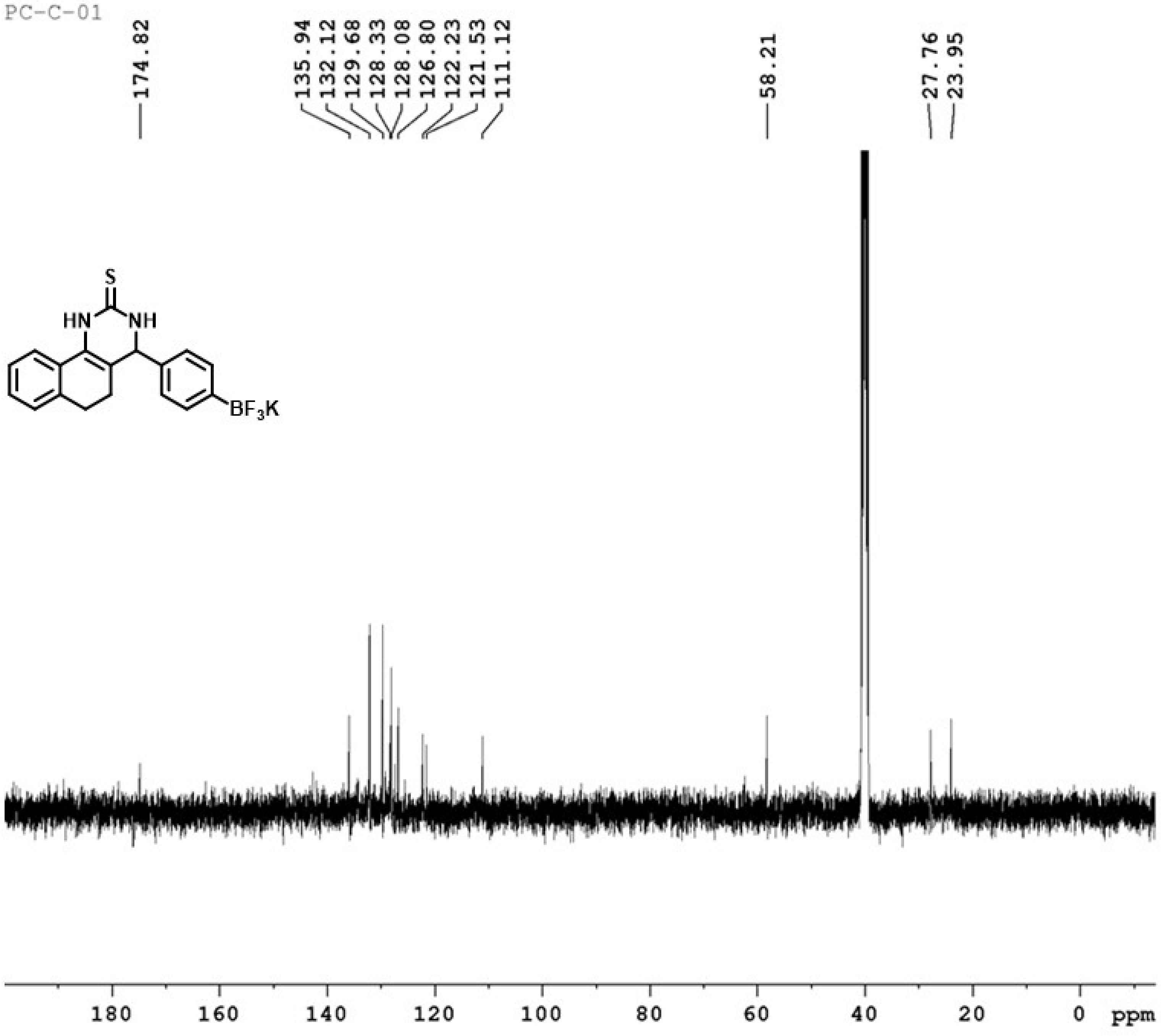

Current Data Parameters

NAME	Aug16-2023
EXPNO	3
PROCNO	1

F2 - Acquisition Parameters

Date_	20230816
Time	13.22 h
INSTRUM	Avance Neo 400 Nanobay
PROBHD	Z163739_0311 (
PULPROG	zgpg30
TD	65536
SOLVENT	DMSO
NS	2048
DS	4
SWH	23809.523 Hz
FIDRES	0.726609 Hz
AQ	1.3762560 sec
RG	101
DW	21.000 usec
DE	6.50 usec
TE	298.0 K
D1	2.00000000 sec
D11	0.03000000 sec
TD0	1
SFO1	100.6266019 MHz
NUC1	13C
P0	2.67 usec
P1	8.00 usec
PLW1	91.95999908 W
SFO2	400.1466006 MHz
NUC2	1H
CPDPRG[2	waltz65
PCPD2	90.00 usec
PLW2	20.98500061 W
PLW12	0.16581000 W
PLW13	0.08340100 W

F2 - Processing parameters

SI	32768
SF	100.6165403 MHz
WDW	EM
SSB	0
LB	1.00 Hz
GB	0
PC	1.40

PC-C-01

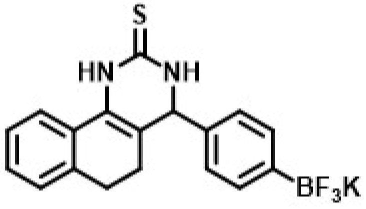

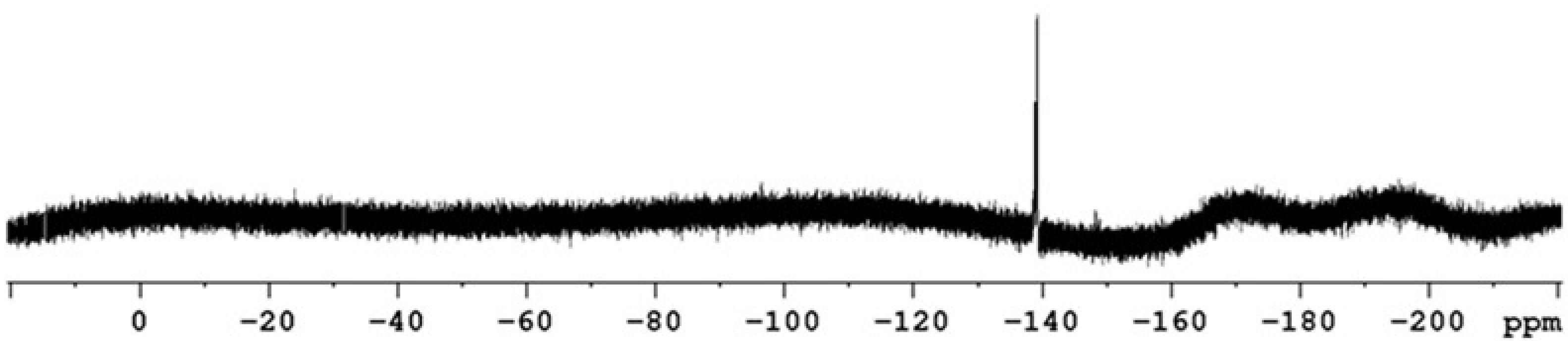

Current Data Parameters	
NAME	Aug16-2023
EXPNO	2
PROCNO	1

F2 - Acquisition Parameters	
Date_	20230816
Time	11.15 h
INSTRUM	Avance Neo 400 Nanobay
PROBHD	Z163739_0311 (
PULPROG	zg
TD	131072
SOLVENT	DMSO
NS	16
DS	4
SWH	90909.094 Hz
FIDRES	1.387163 Hz
AQ	0.7208960 sec
RG	101
DW	5.500 usec
DE	6.50 usec
TE	298.0 K
D1	1.00000000 sec
TD0	1
SFO1	376.4748291 MHz
NUC1	19F
P1	12.00 usec
PLW1	30.18499947 W

F2 - Processing parameters	
SI	65536
SF	376.5124803 MHz
WDW	EM
SSB	0
LB	0.30 Hz
GB	0
PC	1.00
