# Supplementary material for: Design of precision therapeutics for a CKD risk allele by targeting Shroom3-Rock interaction
Source: Nat Commun. 2025 Dec 30;17:1086. doi: 10.1038/s41467-025-67854-7 (PMC12852734; doi:10.1038/s41467-025-67854-7)
Supplement: Supplementary file 5 — Reporting Summary [file 41467_2025_67854_MOESM5_ESM.pdf]

nature portfolio

Corresponding author(s): Dr Madhav C Menon, Dr Cijiang John He and Dr Bhaskar Das

Last updated by author(s): Oct 25, 2025

Reporting Summary

Nature Portfolio wishes to improve the reproducibility of the work that we publish. This form provides structure for consistency and transparency in reporting. For further information on Nature Portfolio policies, see our Editorial Policies and the Editorial Policy Checklist.

Statistics

For all statistical analyses, confirm that the following items are present in the figure legend, table legend, main text, or Methods section.

n/a	Confirmed	
☐	☒	The exact sample size (*n*) for each experimental group/condition, given as a discrete number and unit of measurement
☐	☒	A statement on whether measurements were taken from distinct samples or whether the same sample was measured repeatedly
☐	☒	The statistical test(s) used AND whether they are one- or two-sided<br>*Only common tests should be described solely by name; describe more complex techniques in the Methods section.*
☒	☐	A description of all covariates tested
☐	☒	A description of any assumptions or corrections, such as tests of normality and adjustment for multiple comparisons
☐	☒	A full description of the statistical parameters including central tendency (e.g. means) or other basic estimates (e.g. regression coefficient) AND variation (e.g. standard deviation) or associated estimates of uncertainty (e.g. confidence intervals)
☐	☒	For null hypothesis testing, the test statistic (e.g. *F*, *t*, *r*) with confidence intervals, effect sizes, degrees of freedom and *P* value noted<br>*Give P values as exact values whenever suitable.*
☒	☐	For Bayesian analysis, information on the choice of priors and Markov chain Monte Carlo settings
☒	☐	For hierarchical and complex designs, identification of the appropriate level for tests and full reporting of outcomes
☒	☐	Estimates of effect sizes (e.g. Cohen's *d*, Pearson's *r*), indicating how they were calculated

Our web collection on statistics for biologists contains articles on many of the points above.

Software and code

Policy information about availability of computer code

Data collection	All the IHC staining slides were scanned, images processed using Aperio ImageScope software (v12.3.2), Confocal imaging was done using Stellaris Confocal microscope with Leica LASX software v1.44. For western blot imaging Licor Odyssey Fc imager and Image Studio Lite Ver5.2 were used. qPCR performed using Applied Biosystems 7500 Real-Time PCR System.
Data analysis	Histology staining images and western blots were quantified using NIH ImageJ v2.1.0<br>Bulk RNAseq data analysis: Rsubread package, FeatureCounts version 2.0.0; DESeq2 version 1.44.0; EnrichR analysis of DEGs.<br>The confocal microscopy images were processed using Leica LASX software v1.44.<br>Statistical analyses were performed using GraphPad Prism 10.<br>StepOne software v2.3 was used to analyze qPCR data.

For manuscripts utilizing custom algorithms or software that are central to the research but not yet described in published literature, software must be made available to editors and reviewers. We strongly encourage code deposition in a community repository (e.g. GitHub). See the Nature Portfolio guidelines for submitting code & software for further information.

Data

Policy information about availability of data

All manuscripts must include a data availability statement. This statement should provide the following information, where applicable:

- Accession codes, unique identifiers, or web links for publicly available datasets
- A description of any restrictions on data availability
- For clinical datasets or third party data, please ensure that the statement adheres to our policy

All data are available in the manuscript files. The raw files of Bulk RNA sequencing data are available at the GenBank Bioproject under ID PRJNA1221928 (https://www.ncbi.nlm.nih.gov/bioproject/?term=PRJNA1221928). The analysis code used with RNAseq data has been deposited and publicly available in Github at https://github.com/nrajeevan/Shroom3-Rock_interaction_and_profibrotic_function.git

Research involving human participants, their data, or biological material

Policy information about studies with human participants or human data. See also policy information about sex, gender (identity/presentation), and sexual orientation and race, ethnicity and racism.

Reporting on sex and gender	NA
Reporting on race, ethnicity, or other socially relevant groupings	NA
Population characteristics	NA
Recruitment	NA
Ethics oversight	NA

Note that full information on the approval of the study protocol must also be provided in the manuscript.

Field-specific reporting

Please select the one below that is the best fit for your research. If you are not sure, read the appropriate sections before making your selection.

☒ Life sciences ☐ Behavioural & social sciences ☐ Ecological, evolutionary & environmental sciences

For a reference copy of the document with all sections, see nature.com/documents/nr-reporting-summary-flat.pdf

Life sciences study design

All studies must disclose on these points even when the disclosure is negative.

Sample size	UUO: The mice were sacrificed on day 7 days after UUO (n>=4 mice/experimental group).<br>AAN: The mice were sacrificed on day 63 after AA initial dose (n>=5 mice/experimental group). No sample size calculation was performed prior to experiments using transgenic mice since effect size was unknown. After initial UUO experiments, given an efficacy difference between lines in TIF parameters of 30-50%, we anticipated that >=4 animals in each group ie DMSO or drug would give 90% power for Alpha 0.05.
Data exclusions	In drug testing in Fig 8 one mouse each from D4G and DMSO group died within the first week from surgical complications and were excluded
Replication	We have independently repeated all the experiments 2-3 times. In vivo studies have >= 4 biological replicates in each group.
Randomization	All mice used in the study were age-matched and randomly assigned to each treatment groups. For the drug treatments, littermates were equally divided into the drug and DMSO control group, in a random manner.
Blinding	The investigators were blinded for all Histological analyses including Trichrome/Sirius Red stainings and Immunostainings. Also, the estimations of serum creatinine and urine Albumin:Creatinine levels were also blinded. For all other experiments, the investigators were not blinded, however no subjective assessments were made.

Reporting for specific materials, systems and methods

We require information from authors about some types of materials, experimental systems and methods used in many studies. Here, indicate whether each material, system or method listed is relevant to your study. If you are not sure if a list item applies to your research, read the appropriate section before selecting a response.

Materials & experimental systems

n/a	Involved in the study
☐	☒ Antibodies
☐	☒ Eukaryotic cell lines
☒	☐ Palaeontology and archaeology
☐	☒ Animals and other organisms
☒	☐ Clinical data
☒	☐ Dual use research of concern
☒	☐ Plants

Methods

n/a	Involved in the study
☒	☐ ChIP-seq
☒	☐ Flow cytometry
☒	☐ MRI-based neuroimaging

Antibodies

Antibodies used

Primary antibodies for Western Blot: Anti-MYPT (D6C1) (1:2000; Rabbit mAb #8574, Cell Signaling), anti-phosphoMYPT-T696 (polyclonal)(1:1000; #5163, Cell Signaling), anti-ROCK1 (C8F7)(1:2000; 4035S, Cell Signaling), anti-ROCK2 (D1B1) (1:2000; #9029, Cell Signaling), anti-HSP90 (C45G5) (1:2000; #4877, Cell Signaling), anti-SHROOM3 (polyclonal) (1:500; #SAB3500818, MilliporeSigma), anti-SHROOM3(polyclonal) (1:500; #LS-C679459, LSBio), anti-V5(1:1000; #V8012, MilliporeSigma), anti-Flag(1:500; #A8592, MilliporeSigma), anti-Gapdh (6C5)(1:2000; #AM4300, Invitrogen), anti-β-Actin (AC-15) (1:2000; #A5441, MilliporeSigma), anti-phospho-SMAD3- Ser423/425 (C25A9) (1:1000; #9520, Cell Signaling), anti-SMAD3 (C67H9) (1:1000; #9523, Cell Signaling), anti-Vinculin (7F9) (1:5000; #sc-73614, SCBT). Immunocytochemistry: ROCK1 (polyclonal)(1:100; #PA5-22262; Thermo Scientific). 488 phalloidin (100nM, #PHDG1, Cytoskeleton Inc.) and Anti rabbit Alexafluor-568 secondary antibody (1:300; #A-11036; Invitrogen). Primary antibodies for IHC/IF: Anti-SHROOM3 (polyclonal) (1:500; #LS-C679459, LSBio), anti-Collagen-I (polyclonal)(1:200; #1310-01) and anti-Collagen-III polyclonal)(1:200; 1330-01) from Southern Biotech; anti-FLAG (D6W5B)(1:100; #14793, Cell Signaling), anti-Fibronectin (polyclonal)(1:100; #SAB5700724, MilliporeSigma). Rabbit polyclonal antibody against mouse Megalin (1:1000; anti-MC220 (PMID: 15180987)) and mouse monoclonal antibody against KSP-Cadherin (clone:4F6/F6) (1:1000) kindly provided by Dr Robert Brent Thomson at the Yale Nephrology. Secondary antibodies: Polyclonal HRP-conjugated anti-rabbit (#AP307P, MilliporeSigma) and HRP-conjugated anti-mouse (#AP308P, MilliporeSigma)- 1:8000 to 1:10000. Anti-rabbit Alexafluor-568 secondary antibody (polyclonal)(1:300; #A-11036; Invitrogen) and Alexafluor-594 rabbit anti-Goat (polyclonal)(1:300; #A-11080; Invitrogen), FITC labelled Lotus Tetragonolobus Lectin (1:100; #L32480; Invitrogen), and DAPI (1µg/mL; #D9542; MilliporeSigma).

Validation

All primary antibodies (except the two gifted by Dr Thomson) were commercially purchased (datasheets are available on their websites) and all antibodies have been published in previous publications

Eukaryotic cell lines

Policy information about cell lines and Sex and Gender in Research

Cell line source(s)

HEK293T (CRL-3216) cell line (published in PMID:25437874) was obtained from Dr Cijiang John He (Icahn School of Medicine Mount Sinai), originally obtained from ATCC. mIMCD-3 (CRL-2123) cell line (published in PMID: 38693102) and NIH/3T3 (CRL-1658) cell line were gifted by Dr Stefan Somlo (Yale) and Dr Lloyd Cantley (Yale) respectively, both originally obtained from ATCC. Cryostored early passage aliquots were preferentially expanded for the experiments in the manuscript.

Authentication

The cell lines were not authenticated

Mycoplasma contamination

Molecular testing for Mycoplasma were not done for the cells. The passages of the cells did not show abnormal morphological changes of Mycoplasma infection (elongation, vacuolization, retarded growth and loss of adhesion).

Commonly misidentified lines
(See ICLAC register)

These cell lines are not commonly misidentified

Animals and other research organisms

Policy information about studies involving animals; ARRIVE guidelines recommended for reporting animal research, and Sex and Gender in Research

Laboratory animals

FVB/NJ (expressing Shroom3/TRE and CAGS-rtTA) mice were used as global expression models. FVB/NJ (expressing Shroom3/TRE) and C57BL/6 (expressing rtTAs) mice (Jackson Laboratory) aged 7-9 weeks were crossed for cell-specific overexpression in this work. Where non-transgenic mice were used (Figure 5 & 3, transgene-negative littermate controls were selected from each transgenic strain. All mice were maintained on a 12-hour light and 12-hour dark cycle at the temperature between 68-79 °F and humidity between 30-70% with free access to DOX chow and DOX water before and after surgery/injections. All injury models were initiated in 10-12 week old mice post induction of overexpression by DOX.

Wild animals

This work does not involve any wild animals.

Reporting on sex

Due to difference in susceptibility to Aristolochic acid induced injury between male and female mice (PMID: 39875057), male mice were exclusively used to reduce total numbers of mice required for statistical analysis. For UUO models, both male and female mice were used.

Field-collected samples	No field-collected samples were used in the study as the information is not relevant to this work.
Ethics oversight	Animal protocols were approved by the Yale University Animal Care and Use Committee (IACUC protocol number: 20363)

Note that full information on the approval of the study protocol must also be provided in the manuscript.

Plants

Seed stocks	NA
Novel plant genotypes	NA
Authentication	NA
